# Optimized Chitosan Nanoparticles for Enhanced Ciprofloxacin Delivery and Activity Against Resistant Bacteria

**DOI:** 10.3390/ph19091452

**Published:** 2026-09-14

**Authors:** Lina Alharbi, Ghaida Abalkhail, Fatimah Alabrah, Faisal Alsuwayyid, Raghad R. Alzahrani, Ibrahim Farh, Majed Halwani, Aiman A. Obaidat, Alaa Eldeen B. Yassin

**Affiliations:** 1 College of Pharmacy, King Saud bin Abdulaziz University for Health Sciences, King Abdullah International Medical Research Center, King Abdulaziz Medical City, Ministry of National Guard Health Affairs, Riyadh 11481, Saudi Arabia; linalaafi@gmail.com (L.A.); ghaida.ablk@gmail.com (G.A.); fatimahalobrah@hotmail.com (F.A.); alsuwayyidf@gmail.com (F.A.); farhi@ksau-hs.edu.sa (I.F.); obaidata@ksau-hs.edu.sa (A.A.O.); 2 Saudi Food and Drug Authority, Riyadh 13513, Saudi Arabia; 3 Servier Pharma, Riyadh 21371, Saudi Arabia; 4 Department of Botany and Microbiology, College of Science, King Saud University, Riyadh 11451, Saudi Arabia; raghad.r.alzahrani@gmail.com; 5 King Abdullah International Medical Research Center (KAIMRC-WR), King Saud bin Abdulaziz University for Health Sciences, Ministry of National Guard Health Affairs, King Abdulaziz Medical City, Jeddah 21423, Saudi Arabia; halwanim@kaimrc.med.sa

**Keywords:** ciprofloxacin, chitosan nanoparticles, tripolyphosphate, ion gelation, antibacterial activity

## Abstract

**Background/Objectives**: Ciprofloxacin (CIP) is a fluoroquinolone extensively used in hospital settings for the treatment of bacterial infections; however, this antibiotic requires multiple doses because it has poor absorption, rapid elimination, and is ineffective against resistant bacterial strains. Enhanced delivery efficiency could also be used in the dose-sparing techniques, resulting in better antibiotic efficacy. The aim of this study was to improve CIP-loaded chitosan nanoparticles (NPs) and to evaluate their capacity to enhance the antibacterial response in sensitive and resistant bacterial isolates. **Methods**: CIP-loaded chitosan nanoparticles were fabricated via ionic gelation using sodium tripolyphosphate (TPP) as a crosslinking agent. Formulation parameters, such as CIP concentration and polymer-to-crosslinker ratios, were optimized. The obtained nanoparticles were evaluated for particle size, polydispersity index, zeta potential, entrapment efficiency, morphology, stability, and in vitro release studies. Antibacterial efficacy was evaluated by determining minimum inhibitory concentration (MIC) and minimum bactericidal concentration (MBC) values against standard and ciprofloxacin-resistant clinical isolates. **Results**: Successful optimization of these formulations allowed the preparation of stable nanoparticles that ranged from 38.39 to 115.07 nm, highlighting the role of both the formulation composition and polymer-to-crosslinker ratios on the size of the nanoparticles. The formulation exhibits a drug entrapment efficiency of 84.1% and uniform particulate size (38.39 ± 0.63 nm), which were optimized due to the polymer and crosslinker ratios. These nanoparticles showed a slow release of the drug over 220 h, and minimal size instability was noted after 28 days. Encapsulation of CIP resulted in enhanced antibacterial activity, yielding 2–4-fold reductions in MIC and MBC values against methicillin-resistant *Staphylococcus aureus* and *Pseudomonas aeruginosa* resistant strains, compared with free CIP. **Conclusions**: Optimized CIP-loaded chitosan nanoparticles demonstrated improved antibacterial efficacy against resistant strains and good drug delivery properties. These findings highlight the potential of chitosan-based nanocarrier systems to improve the performance of CIP and antibiotic delivery dose-sparing antibiotic strategies.

## 1. Introduction

Chitosan (CS), the second most abundant polymer in nature, is prepared by deacetylation of chitin obtained from shellfish through treatment with a hot concentrated solution of sodium hydroxide [1,2]. Due to its distinctive cationic nature, CS exhibits several advantageous physicochemical and biological properties such as biocompatibility, biodegradability, mucoadhesion, and permeability enhancement [3,4,5,6]. Additionally, CS has been reported to exhibit intrinsic antimicrobial activity against a wide range of microorganisms, including resistant strains such as *Escherichia coli*, *Staphylococcus aureus*, and methicillin-resistant *S. aureus* (MRSA), which is attributed to its polycationic interaction with negatively charged microbial membranes leading to cell disruption and leakage [7,8,9,10,11,12]. Recent advances in nanotechnology have further improved CS’s performance as a nanocarrier, particularly in nanoparticle (NP) systems produced by ionic gelation, which enables mild and efficient encapsulation of drugs without harsh solvents or conditions [13,14].

CIP, a second-generation fluoroquinolone, remains one of the most widely used broad-spectrum antibiotics for treating diverse bacterial infections including those of the urinary tract, respiratory system, skin, bones, and gastrointestinal tract [15,16,17]. However, despite its efficacy, CIP suffers from pharmacokinetic limitations such as incomplete oral absorption, and relatively rapid systemic elimination, which may limit prolonged maintenance of effective drug concentrations [18,19]. Such drawbacks often result in suboptimal therapeutic concentrations, increased side effects, and accelerated emergence of resistant bacterial strains [20]. Therefore, advanced formulation strategies capable of improving CIP delivery, prolonging drug release, maintaining effective antimicrobial exposure, and enhancing antibacterial activity are highly desirable.

Nanoparticulate delivery systems have emerged as a versatile approach for improving the therapeutic performance of conventional antibiotics by enhancing their pharmacokinetics, reducing toxicity, and facilitating intracellular delivery [21,22,23,24]. Recent advances further demonstrate the importance of nanocarrier architecture in determining loading and delivery performance. For example, Dong et al. developed biomineralized copper carbonate nanoparticles with high macromolecular loading capacity, pH-responsive drug release, and enhanced intracellular delivery, highlighting the versatility of rationally designed nanocarriers for improving therapeutic payload delivery [25]. Among these, polymeric nanoparticles—particularly those based on biopolymers like CS—are especially promising due to their ability to encapsulate hydrophilic and hydrophobic agents, protect labile drugs from degradation, and enable targeted and controlled drug release [26,27,28]. Previous studies demonstrated that CS-based nanoparticles effectively enhance the antibacterial activity of encapsulated drugs. For instance, Zaki and Hafez [29] reported improved intracellular delivery and efficacy of ceftriaxone against *Salmonella typhimurium* using CS–TPP (tripolyphosphate) nanoparticles, while Sobhani et al. [30] observed significant MIC reduction in CIP-loaded CS nanoparticles against *E. coli* and *S. aureus*. Similarly, CS-coated albumin nanoparticles loaded with colistin exhibited potent antimicrobial and antibiofilm activity against multidrug-resistant Gram-negative bacteria [31].

Beyond enhanced drug delivery, chitosan-based nanocarriers can also help mitigate bacterial resistance by multiple mechanisms including improved bacterial membrane interaction, inhibition of efflux pumps, and sustained drug exposure [32,33,34]. Recent investigations into hybrid polymeric–lipid and metallic nanocarriers further highlight the role of nanotechnology in combating multidrug resistance through synergistic effects, biofilm penetration, and modulation of bacterial quorum sensing [35,36,37,38]. Such multifactorial action offers a valuable platform for repurposing existing antibiotics like CIP with improved efficacy and safety.

Accordingly, this study aimed to optimize the encapsulation of CIP within CS-nanoparticles possessing favorable physicochemical and biopharmaceutical properties, with the goal of enhancing antibacterial efficacy and overcoming bacterial resistance.

## 2. Results

Table 1 presents the composition of the nine CS nanoparticle formulations developed to optimize particle size and CIP encapsulation efficiency (EE%). Various concentrations of CIP, CS, and TPP were systematically investigated through a series of preliminary experiments to identify the most favorable formulation parameters. Among the tested formulations, Formulation 8 (F8), containing 0.15 mg/mL CIP, 0.3 mg/mL CS, and 0.5 mg/mL TPP, exhibited the most desirable physicochemical characteristics. This formulation produced nanoparticles with a mean particle size of 38.39 ± 0.63 nm and an exceptionally low polydispersity index (PDI = 0.011), indicating a highly homogeneous particle population. In addition, F8 achieved an encapsulation efficiency of 84.1%, demonstrating its strong capacity to efficiently incorporate and retain CIP within the chitosan nanoparticle matrix. These findings suggest that F8 represents the optimal formulation among those evaluated and provides a promising platform for enhancing CIP delivery.

### 2.1. Characterization of the Nanoparticles

#### 2.1.1. Particle Size

**Figure 1:**
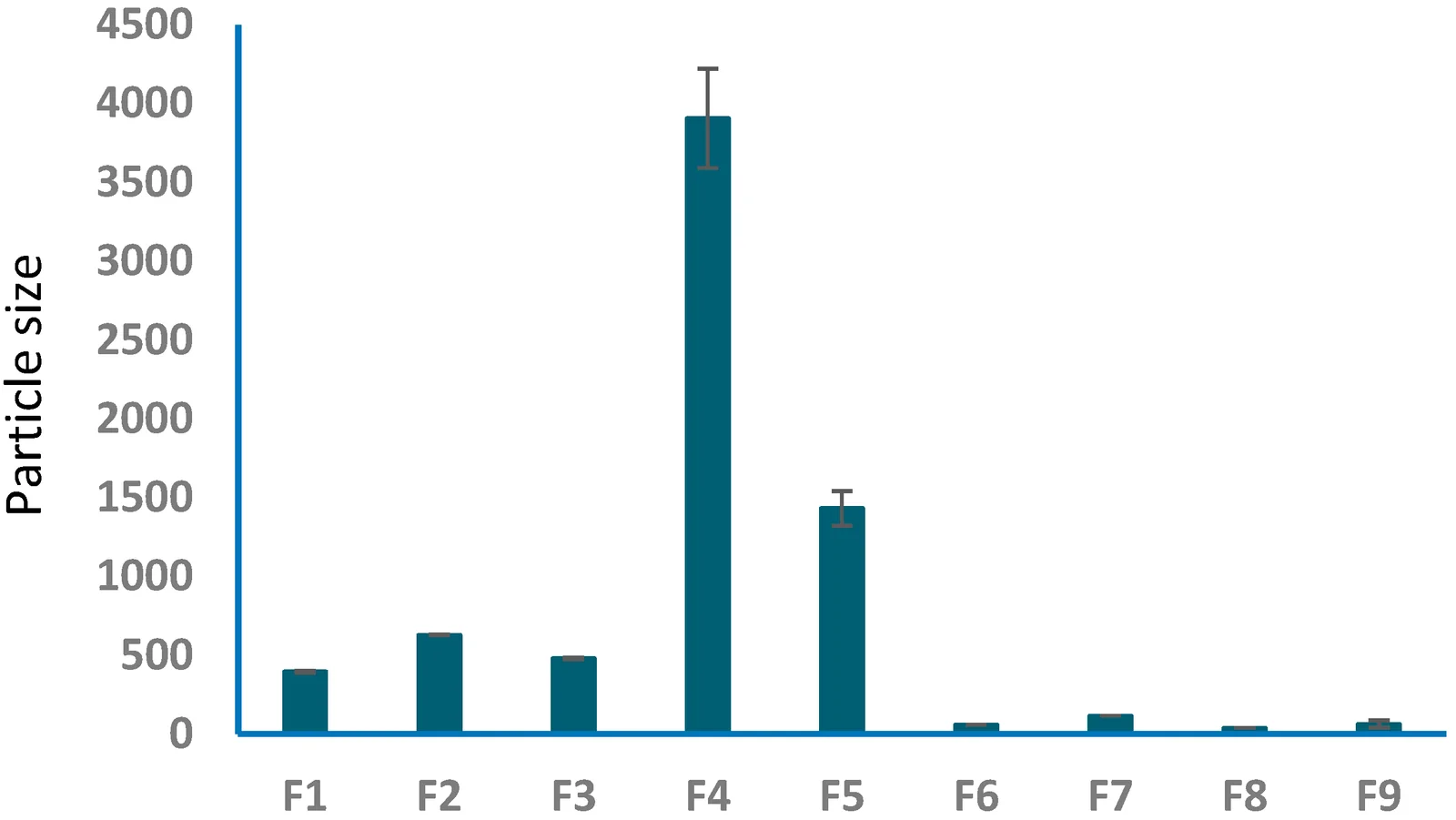
Mean particle sizes for all CIP-loaded CS nanoparticles formulations.

Figure 1 illustrates the effect of formulation composition and processing parameters on the particle size of ciprofloxacin (CIP)-loaded chitosan (CS) nanoparticles. Considerable variation in particle size was observed among the tested formulations, indicating the critical role of formulation variables in determining nanoparticle characteristics. Formulation 1 (F1), prepared without sonication, produced nanoparticles with a mean particle size of 395.34 ± 6.09 nm. Increasing the formulation complexity in F2 resulted in a larger particle size of 628.50 ± 3.79 nm, which may be attributed to insufficient particle dispersion and increased aggregation during nanoparticle formation. Similarly, F3, prepared using a CS-to-TPP ratio of 2:1, generated nanoparticles with an intermediate particle size of 479.03 ± 7.05 nm.

In contrast, F4 and F5 exhibited markedly larger particle sizes of 3907.39 ± 315.35 nm and 1431.77 ± 109.65 nm, respectively. The exceptionally large mean diameter and high standard deviation observed for F4 indicate extensive particle aggregation and poor formulation reproducibility, while the large particle size of F5 suggests inadequate stabilization of the nanoparticle system.

A substantial reduction in particle size was achieved in F 6–9, which produced nanoparticles within the nanoscale range (38.39–115.07 nm). This improvement demonstrates the importance of optimizing the concentrations of CS, TPP, and CIP to promote efficient ionic gelation and particle stabilization. Among all formulations, F8 produced the smallest nanoparticles (38.39 ± 0.63 nm) and exhibited an exceptionally low polydispersity index (PDI = 0.011), indicating a highly homogeneous particle population. Although F9 also generated relatively small nanoparticles (63.03 ± 23.41 nm), its larger standard deviation and higher PDI reflected greater variability in particle size distribution.

The superior performance of F8 can be attributed to the optimized balance between polymer and crosslinker concentrations, which facilitated controlled nanoparticle formation while minimizing aggregation. Collectively, these findings identify F8 as the optimal formulation, providing the smallest particle size, highest homogeneity, and most favorable physicochemical characteristics for further development as a ciprofloxacin-loaded nanoparticulate delivery system.

#### 2.1.2. Polydispersity Index (PDI) Analysis

**Figure 2:**
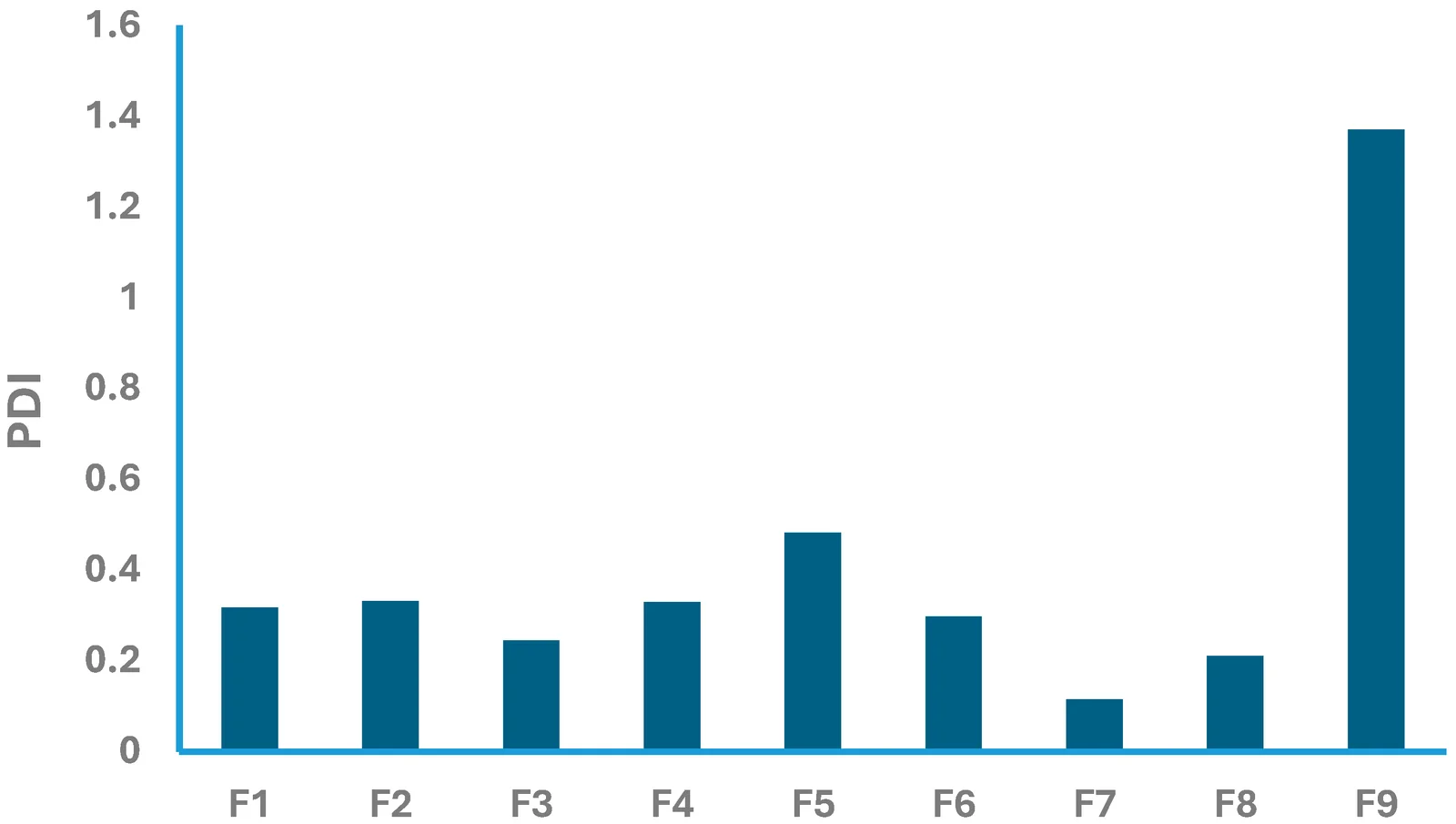
Polydispersity index (PDI) for all CIP-loaded CS nanoparticles formulations.

The polydispersity index (PDI) is a critical parameter used to assess the uniformity of particle size distribution within nanoparticle formulations. Lower PDI values indicate a more homogeneous particle population and are generally associated with improved colloidal stability, reproducibility, and predictable drug delivery performance. The PDI values of the nine ciprofloxacin-loaded chitosan (CIP-loaded CS) nanoparticle formulations are presented in Table 2 and Figure 2.

Formulations F1 and F2 exhibited moderate size distributions, with PDI values of 0.317 ± 0.004 and 0.332 ± 0.002, respectively, suggesting acceptable but relatively broad particle size variability. Formulations F3 and F6 showed improved homogeneity, with PDI values of 0.245 ± 0.013 and 0.298 ± 0.001, respectively. Similar behavior was observed for F4 (0.330 ± 0.011), despite its exceptionally large particle size, indicating that the particles were relatively uniform but extensively aggregated. In contrast, F5 exhibited a considerably higher PDI (0.482 ± 0.017), reflecting a broad size distribution and poor formulation uniformity.

Among all formulations, F8 demonstrated the lowest PDI value (0.011 ± 0.001), indicating an exceptionally narrow particle size distribution and a highly homogeneous nanoparticle population. This result is consistent with its small particle size and further confirms the superior physicochemical characteristics of this formulation. Formulation F7 also exhibited good uniformity (PDI = 0.115 ± 0.037), whereas F9 displayed the highest PDI value (1.37 ± 0.23), indicating extreme heterogeneity and the presence of multiple particle populations or severe aggregation, which could adversely affect formulation stability, reproducibility, and drug delivery efficiency.

Overall, the PDI results demonstrate the strong influence of formulation composition on nanoparticle uniformity. The combination of the smallest particle size and the lowest PDI observed for F8 highlights its superior stability and homogeneity compared with the other formulations, supporting its selection as the optimal ciprofloxacin-loaded chitosan nanoparticle formulation for further characterization and biological evaluation.

#### 2.1.3. Zeta Potential Analysis

**Figure 3:**
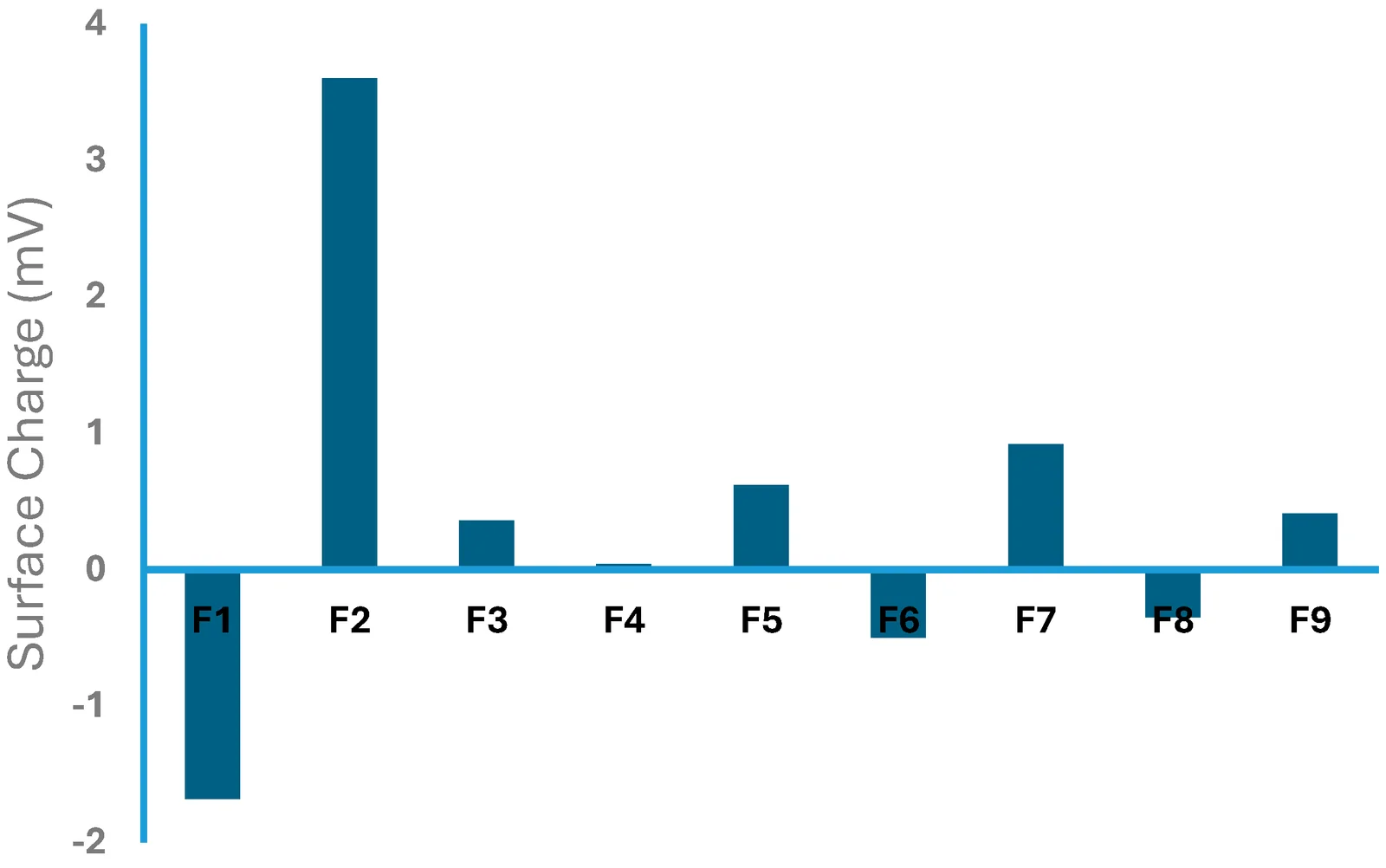
Zeta potential for all CIP-loaded CS nanoparticles formulations.

The zeta potential results (Figure 3) revealed relatively low surface charge values across all ciprofloxacin-loaded chitosan (CIP-loaded CS) nanoparticle formulations, ranging from −1.68 ± 1.13 mV to 3.60 ± 3.33 mV. Formulations F2 and F7 exhibited the highest positive zeta potentials, while F1 showed the most negative value. Most formulations displayed near-neutral surface charges, indicating limited electrostatic stabilization and a potential tendency toward particle aggregation. Among the tested formulations, F8 exhibited a slightly negative zeta potential (−0.35 ± 0.65 mV), which, together with its small particle size and exceptionally low PDI, suggests that factors other than electrostatic repulsion, such as steric stabilization and efficient crosslinking, contributed to its favorable stability. Overall, the findings highlight the importance of surface charge in nanoparticle stability and suggest that optimization of formulation parameters is essential to achieve a balance between colloidal stability and drug delivery performance.

#### 2.1.4. Particle Physical Stability

**Figure 4:**
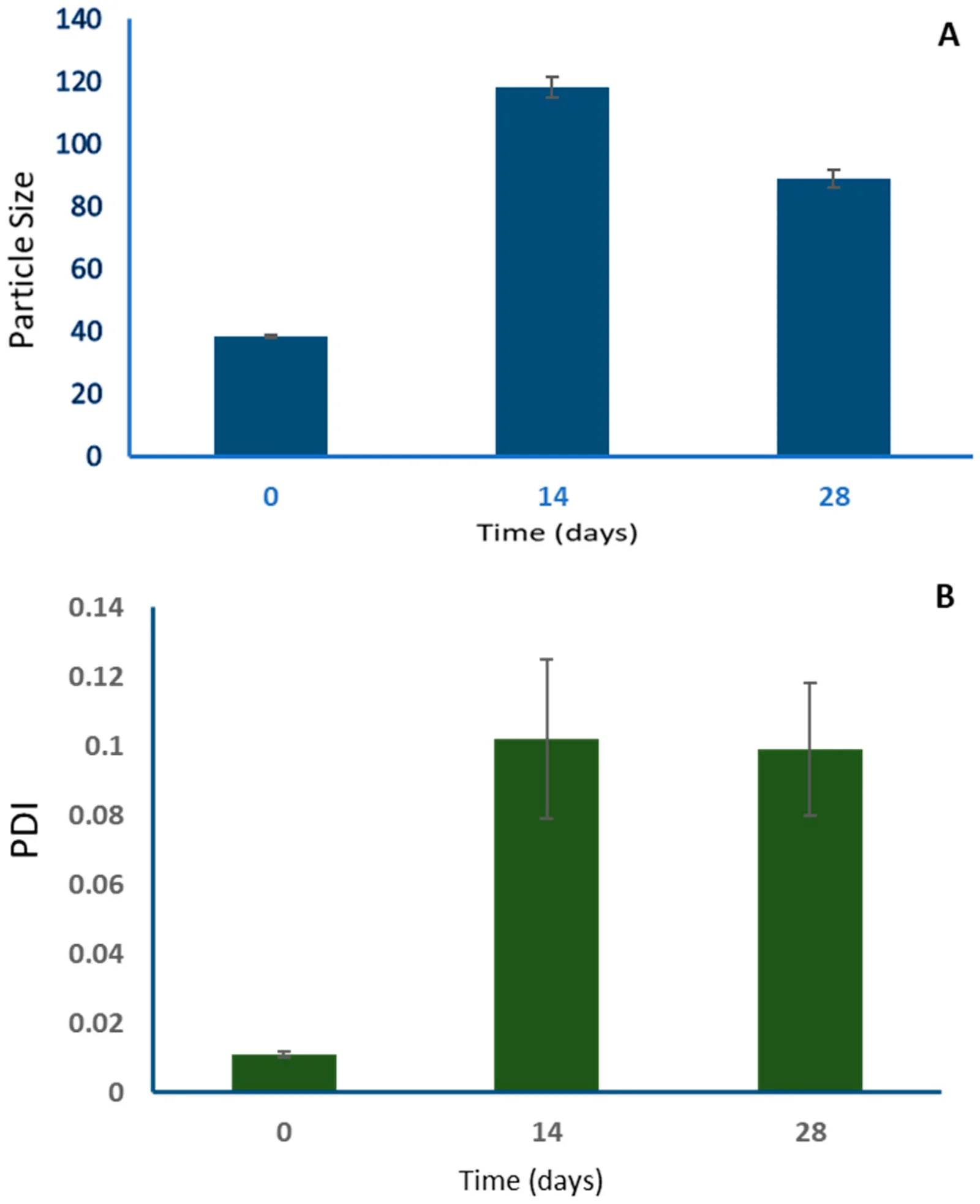
Particle size stability of the optimized CIP-loaded chitosan nanoparticle formulation (F8) during 28 days of storage. (**A**) represents the change in particle size and (**B**) represents the changes in PDI.

Figure 4 illustrates the physical stability of the optimized CIP-loaded chitosan nanoparticle formulation (F8) over a 28-day storage period, based on changes in particle size and polydispersity index (PDI). At Day 0, F8 exhibited a mean particle size of 38.39 ± 0.63 nm with a very low PDI of 0.011 ± 0.001, confirming the formation of highly uniform nanoparticles with a narrow size distribution.

After 14 days of storage, the particle size increased to 118.30 ± 3.24 nm, accompanied by an increase in PDI to 0.102 ± 0.023. This increase may be attributed to nanoparticle swelling, hydration, or limited particle rearrangement during storage and reconstitution following freeze-drying. However, the formulation remained within the nanoscale range, and the PDI value remained low, indicating that the particle population retained acceptable homogeneity without evidence of extensive aggregation.

By Day 28, the particle size decreased to 88.88 ± 2.71 nm, while the PDI remained nearly unchanged at 0.099 ± 0.019. This reduction in particle size after the initial increase suggests partial stabilization of the nanoparticle system following the early swelling or rehydration phase. The maintenance of low PDI values throughout the study indicates that the formulation preserved a relatively narrow size distribution over time.

Overall, although F8 showed moderate changes in particle size during storage, it maintained nanoscale dimensions and consistently low PDI values over the 28-day period. These findings demonstrate that the optimized formulation retained acceptable physical stability, with no indication of irreversible aggregation or marked loss of homogeneity, supporting its suitability as a stable ciprofloxacin-loaded chitosan nanoparticle delivery system.

#### 2.1.5. Entrapment Efficiency

Table 2 presents the entrapment efficiencies (EE%) of formulations F6–F9 along with their physicochemical properties. These formulations were selected for EE% evaluation as they exhibited the smallest particle sizes among all prepared formulations, indicating successful nanoparticle formation. Entrapment efficiency varied across formulations, reflecting differences in drug loading capacity. Formulation F6 showed a moderate EE% of 59.1%, whereas F8 achieved an EE% of 84.1%, and F9 demonstrated the highest EE% at 88.0%.

EE% values were calculated using corrected initial CIP concentrations and experimentally measured entrapped drug amounts, with appropriate dilution factor corrections. Overall, these findings indicate that formulation parameters substantially influence drug encapsulation efficiency, with F8 and F9 providing enhanced drug loading and demonstrating suitability for further therapeutic applications.

#### 2.1.6. Particles’ Morphology

**Figure 5:**
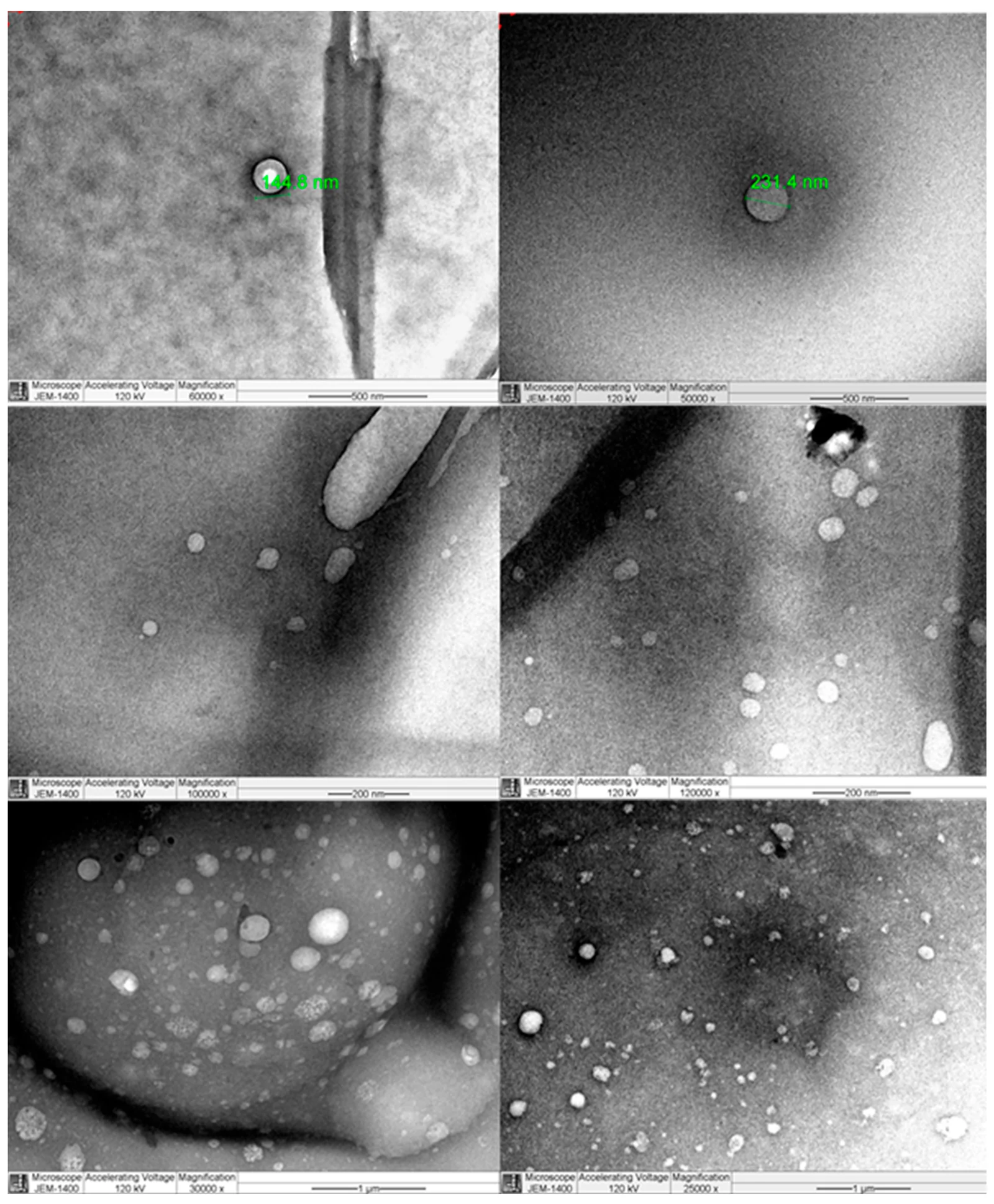
TEM images of different CIP-loaded CS nanoparticles formulation at different magnification powers.

Transmission electron microscopy (TEM) was used to examine the morphology and structural characteristics of ciprofloxacin-loaded chitosan (CIP-loaded CS) nanoparticles. Representative TEM images are shown in Figure 5. The micrographs revealed predominantly spherical nanoparticles with smooth surfaces and uniform dispersion. Nanoparticles from formulations F8 and F9 appeared well defined and homogeneously distributed, confirming the reproducibility of the synthesis method in producing size-controlled particles. The contrast observed within the nanoparticles suggests compact internal structures, which may be attributed to effective CIP–CS interactions and ionic crosslinking, contributing to nanoparticle stability and controlled drug release behavior. Overall, TEM analysis supports the physicochemical characterization results, confirming the successful formation of uniform and stable CIP-loaded CS nanoparticles with morphologies suitable for efficient drug delivery applications.

#### 2.1.7. In Vitro Release Profile of Ciprofloxacin from Nanoparticles

**Figure 6:**
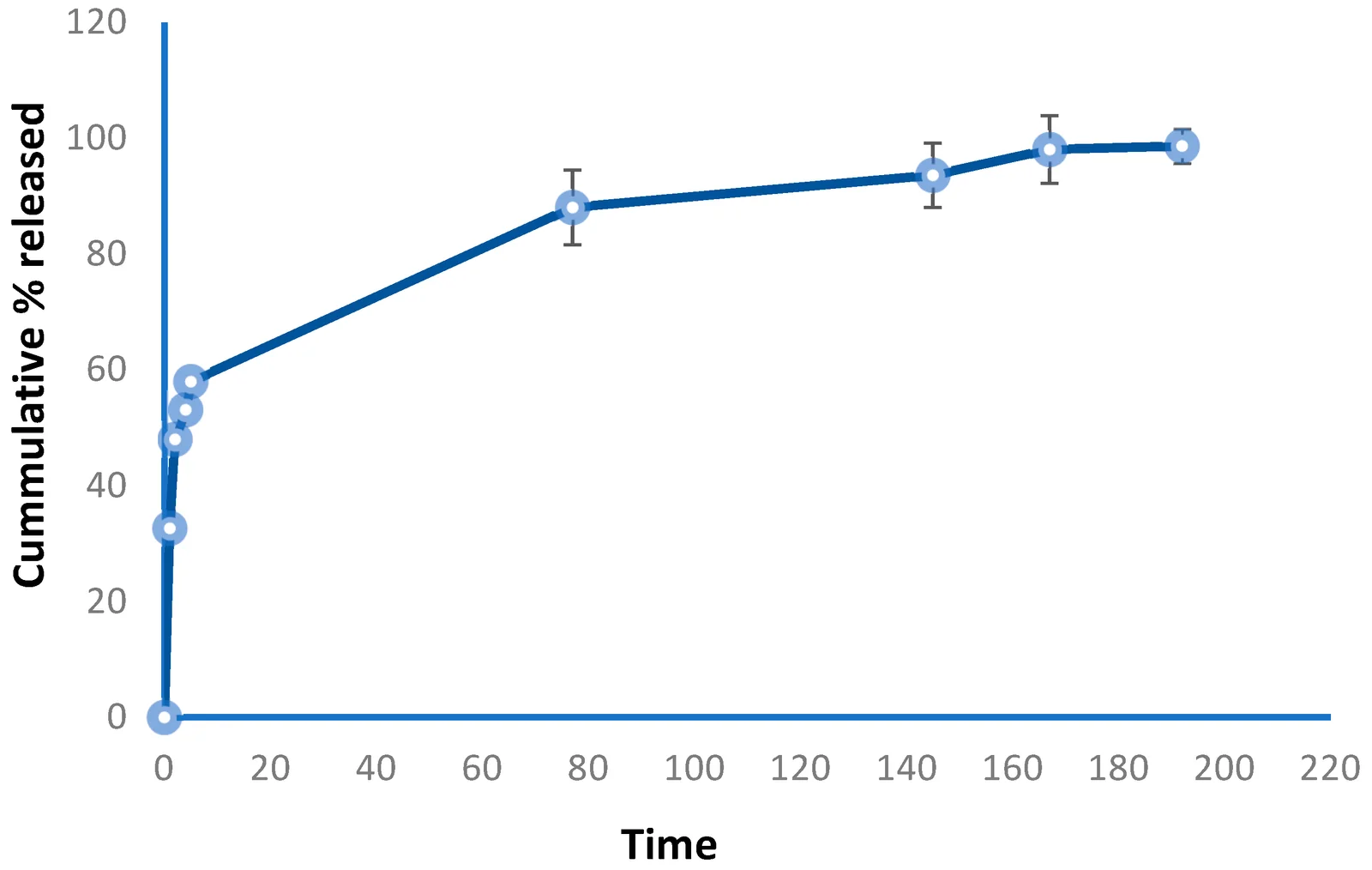
In vitro Release Profile of CIP from CS Nanoparticles of F8.

The in vitro release profile of ciprofloxacin from chitosan nanoparticles was evaluated using the dialysis bag method (Figure 6). The release pattern exhibited a characteristic biphasic behavior, consisting of an initial burst release followed by a prolonged sustained-release phase. Approximately 58% of the encapsulated ciprofloxacin was released within the first 6 h, indicating rapid liberation of drug molecules located near or adsorbed onto the nanoparticle surface. Such an initial burst release may facilitate the rapid attainment of therapeutic drug concentrations at the site of infection.

Following the burst phase, the release rate gradually decreased, with cumulative drug release reaching approximately 88% after 72 h and continuing in a controlled manner to approximately 99% after 192 h. This sustained-release phase is likely governed by the diffusion of ciprofloxacin through the hydrated chitosan matrix, along with gradual polymer swelling and erosion processes that regulate drug transport from the nanoparticle core.

The observed biphasic release behavior is advantageous for antibacterial drug delivery, as it combines an immediate release component capable of providing prompt antimicrobial activity with a prolonged release phase that maintains therapeutic drug levels over an extended period. Such a release profile may reduce dosing frequency, improve patient compliance, and enhance the overall effectiveness of ciprofloxacin therapy against persistent bacterial infections.

Analysis of the release kinetics parameters, displayed in Table 3, demonstrated that CIP release from the optimized F8 formulation was best described by the Higuchi model (R^2^ = 0.979), compared with the Hixson–Crowell (R^2^ = 0.945) and zero-order (R^2^ = 0.930) models. The superior Higuchi fit indicates that CIP release was predominantly diffusion-controlled, most likely involving diffusion of the drug through the hydrated, ionically crosslinked CS–TPP matrix. The relatively high correlation with the Hixson–Crowell model suggests that changes in particle dimensions or gradual matrix relaxation/dissolution may additionally contribute to the release process. Moreover, the Korsmeyer–Peppas exponent (n = 0.334), which is below the characteristic Fickian value for spherical systems, indicates a quasi-Fickian diffusion mechanism. Collectively, these findings suggest that diffusion through the hydrated polymeric network is the principal mechanism governing the prolonged release of CIP from F8, with matrix-related changes playing a secondary role.

#### 2.1.8. Antibacterial Activity of Ciprofloxacin-Loaded Chitosan Nanoparticles (CIP-CS NPs)

The antibacterial efficacy of CIP-CS NPs was evaluated by determining the minimum inhibitory concentration (MIC) and minimum bactericidal concentration (MBC) against both susceptible reference strains and CIP-resistant clinical isolates, in accordance with EUCAST guidelines. The results are summarized in Table 4. For the susceptible reference strain *Escherichia coli* ATCC 25922, free CIP exhibited MIC and MBC values of 0.06 mg/L and 0.125 mg/L, respectively, whereas CIP-CS NPs achieved lower values of <0.06 mg/L for both, indicating enhanced antibacterial potency upon nanoparticle encapsulation. Against methicillin-resistant *Staphylococcus aureus* (MRSA-60), free CIP showed limited activity (MIC = 16 mg/L; MBC = 32 mg/L), while CIP-CS NPs significantly reduced these values to 4 mg/L and 8 mg/L, respectively. A similar improvement was observed for MRSA-145, where MIC/MBC values decreased from 8/16 mg/L for free CIP to 4/8 mg/L for CIP-CS NPs, confirming enhanced activity against resistant Gram-positive strains.

For *Pseudomonas aeruginosa* resistant isolates PA-10 and PA-135, CIP-CS NPs demonstrated marked improvements in antibacterial efficacy. In PA-10, MIC/MBC values decreased from 32/64 mg/L for free CIP to 16/16 mg/L for CIP-CS NPs, while in PA-135, values were reduced from 64/128 mg/L to 32/32 mg/L. Even for the susceptible *P. aeruginosa* PA-598 strain, nanoparticle encapsulation further enhanced activity, lowering MIC/MBC values from 0.25/0.5 mg/L to 0.125/0.25 mg/L. Overall, CIP-CS nanoparticles consistently exhibited lower MIC and MBC values compared with free CIP across both susceptible and resistant strains. This enhanced antibacterial performance may be attributed to controlled drug release, improved bacterial penetration, and sustained local CIP concentrations, supporting the potential of CIP-CS nanoparticles as an advanced formulation for overcoming antibiotic resistance.

## 3. Discussion

CS nanoparticles have been produced using various preparation techniques and have found broad applications, particularly in medicine, as well as in other fields such as agriculture, cosmetics, and wastewater treatment [1,39]. Their physicochemical and functional characteristics may differ markedly depending on the fabrication method and any applied surface modifications, thereby enabling their use in diverse applications [40,41]. The present study demonstrated that formulation variables exerted a profound influence on the physicochemical characteristics of CIP-loaded CS nanoparticles prepared by ionic gelation. Particle size, size distribution, and surface charge are among the most critical determinants of nanoparticle performance, as they directly affect colloidal stability, cellular uptake, drug release behavior, and antimicrobial efficacy [42,43]. The wide variation in particle size observed among formulations (38.39–3907.39 nm) highlights the sensitivity of the ionic gelation process to the concentrations and ratios of CS, TPP, and CIP.

The formation of CS nanoparticles is primarily governed by electrostatic interactions between the protonated amino groups of CS and the negatively charged phosphate groups of TPP during the ionic gelation process. However, nanoparticle formation is influenced by several interrelated polymeric and physicochemical variables, including CS to crosslinker ratio, CS molecular weight and degree of deacetylation, solution pH, and polymer concentration [44,45]. The physicochemical compatibility between CIP and CS in nanoparticle formulations has been extensively investigated and confirmed in previous studies using complementary analytical techniques, particularly DSC and FTIR [30,46,47]. An optimal balance between the polymer and crosslinker promotes the formation of compact, uniformly crosslinked nanoparticles, whereas deviations from this balance may lead to excessive intermolecular interactions, particle aggregation, and increased particle growth. This behavior was evident in formulations F4 and F5, which exhibited exceptionally large particle sizes and broad size distributions, indicating uncontrolled crosslinking and poor colloidal stability. The influence of the CS ratio on nanoparticle characteristics has been extensively documented. Des Bouillons-Gamboa et al. [44] demonstrated that reducing the CS ratio from 6:1 to 3:1 progressively decreased the hydrodynamic diameter from approximately 202 to 178 nm, reflecting enhanced crosslinking efficiency at higher TPP concentrations. However, a further reduction to a 2:1 ratio resulted in pronounced flocculation and precipitation due to excessive charge neutralization of the chitosan chains, leading to instability of the colloidal system [44]. Similarly, Calvo et al. [45] systematically investigated the ionic gelation process and reported that nanoparticle size and surface charge were strongly influenced by CS molecular weight, chitosan concentration, solution pH, and particularly the CS-to-TPP ratio. Cai et al. [48] reported CS nanoparticles prepared by ionic gelation with particle sizes ranging from 198.7 to 373.4 nm, while Gan et al. [49] reported CS nanoparticles with particle sizes between 100 and 250 nm for gene delivery. Similar observations were reported by Liu and Gao [50], who demonstrated that increasing TPP concentration generally promotes the formation of smaller and more compact nanoparticles owing to a higher degree of ionic crosslink. The authors demonstrated that careful control of these formulation parameters enabled the production of CS-TPP nanoparticles with predictable low particle sizes and high positive surface charges, emphasizing the critical role of the polymer-crosslinker balance in determining nanoparticle properties. The present findings are generally consistent with these observations, as formulations F4 and F5 exhibited marked particle enlargement and aggregation, suggesting that the polymer-crosslinker balance had moved beyond the optimal range required for stable nanoparticle formation. These studies collectively confirm that nanoparticle size is highly dependent on formulation composition and crosslinking conditions.

In contrast, formulations F6–F9 produced nanoparticles within the nanoscale range, indicating that optimization of formulation parameters significantly improved nanoparticle formation. Among these formulations, F8 exhibited the smallest particle size (38.39 ± 0.63 nm), which is considerably smaller than the particle sizes commonly reported for CS nanoparticles prepared by ionic gelation. For example, Wu et al. [51] prepared TPP-crosslinked CS nanoparticles with particle sizes generally exceeding 100 nm, while maintaining acceptable colloidal stability and encapsulation properties. Likewise, El-Naggar et al. [52] reported CS nanoparticles with particle sizes between 6.92 and 10.10 nm using a green synthesis approach, whereas conventionally prepared ionic-gelation nanoparticles typically ranged from 166 to 1230 nm depending on formulation conditions. Furthermore, Des Bouillons-Gamboa et al. [44] emphasized that nanoparticle size, PDI, and zeta potential are strongly influenced by the molecular weight and degree of deacetylation of CS. Previous studies have shown that higher molecular-weight CS generally produces larger particles with broader size distributions, whereas lower molecular-weight CS facilitates the formation of smaller and more homogeneous nanoparticles [53,54]. Solution pH is another important factor governing the extent of ionic crosslinking. Under acidic conditions, protonation of the amino groups of CS promotes their electrostatic interaction with the negatively charged phosphate groups of TPP, whereas variations in pH can influence the ionization of both components and consequently affect nanoparticle size and surface charge [45]. In the present study, the acidic conditions were standardized by using 5 mol% acetic acid, thereby maintaining consistent protonation conditions during nanoparticle preparation. These factors may partially explain the differences observed between the present study and previously published reports. Interestingly, the optimized formulation F8 in the present study produced nanoparticles with a mean particle size of only 38.39 ± 0.63 nm, which is substantially smaller than the particle sizes reported by Des Bouillons-Gamboa et al. [44], Calvo et al. [45], and most previously reported CS nanoparticles prepared by ionic gelation [48,49,55]. The remarkable size reduction achieved in F8 may be attributed to the combined effects of optimized CS, TPP, and CIP concentrations together with the applied processing conditions, which promoted efficient nucleation and restricted particle growth. Nanoparticles within this size range are expected to possess a larger specific surface area and enhanced interaction with bacterial cells, potentially improving drug delivery efficiency and antibacterial activity.

The PDI results further confirmed the critical role of formulation optimization in achieving homogeneous nanoparticle populations. PDI values below 0.3 are generally considered indicative of acceptable monodisperses in nanoparticle systems, whereas values exceeding 0.5 suggest substantial heterogeneity. The remarkably low PDI observed for F8 (0.011 ± 0.001) indicates an exceptionally narrow particle size distribution and highly uniform particle population. Such a low PDI is rarely reported for CS nanoparticles and reflects excellent control over the ionic gelation process. Des Bouillons-Gamboa et al. [44] reported PDI values ranging from 0.472 to 0.563 for chitosan nanoparticles prepared at different CS ratios, indicating moderately broad size distributions. Likewise, Rampino et al. [55] reported PDI values typically ranging between 0.2 and 0.5 depending on formulation conditions and storage stability. Fan et al. [56] successfully prepared monodisperse low-molecular-weight CS/TPP nanoparticles with a mean particle size of 138 nm, a zeta potential of +35 mV, and a low PDI of 0.026, demonstrating the importance of carefully controlling ionic-gelation parameters to achieve uniform nanoparticle populations. Consistent with these observations, the optimized F8 formulation exhibited an even lower PDI of 0.011 ± 0.001, indicating a highly homogeneous particle population and effective control of nanoparticle nucleation and growth under the selected formulation and processing conditions. This high degree of uniformity may enhance formulation reproducibility and contribute to more predictable drug-release and biological performance. Encapsulation efficiency (EE) is another important parameter influencing the therapeutic potential of nanoparticle systems. The EE values obtained in the present study ranged from 48.3% to 88%, with F8 exhibiting a high EE of 84.1%. Such efficient drug entrapment can be attributed to strong electrostatic interactions between CIP and the CS matrix, together with the formation of a densely crosslinked nanoparticle network that effectively retains the drug during nanoparticle preparation. Comparable EE values have been reported for CS-based nanocarriers loaded with bioactive compounds, where ionic gelation facilitated efficient incorporation of therapeutic agents through physical entrapment and molecular interactions. Cai et al. [48] reported encapsulation efficiencies ranging from approximately 50% to 85% depending on formulation composition and loading conditions. The high EE observed for F8 therefore confirms the suitability of the selected formulation parameters for effective CIP incorporation. High encapsulation efficiency is particularly beneficial for drug delivery, as it enables lower drug volumes and enhances dose utilization, a critical factor for reducing pill burden and improving patient compliance [38,57]. The high entrapment efficiency observed for F8 (84.1%) is likely attributable to the combined effects of non-covalent drug–polymer interactions and the architecture of the ionically crosslinked CS–TPP matrix. Ionic gelation between protonated CS amino groups and negatively charged TPP phosphate groups generates a compact three-dimensional polymeric network capable of physically restricting CIP diffusion and loss during nanoparticle formation. In addition, the carboxyl and carbonyl groups of CIP can participate in hydrogen bonding with the hydroxyl and amino groups of CS, while the ionization of CIP under the preparation conditions may permit additional ionic associations involving CIP, CS, and TPP. Collectively, these electrostatic and hydrogen-bonding interactions, together with physical confinement within the densely crosslinked matrix, can enhance drug retention. The optimized CS balance in F8 may therefore have produced a sufficiently compact and homogeneous network to maximize CIP incorporation while limiting its diffusion into the external aqueous phase.

The zeta potential measurements revealed relatively low surface charge values for all formulations, ranging from −1.68 to +3.60 mV. These values are considerably lower than those commonly reported for chitosan nanoparticles, which frequently exhibit positive zeta potentials exceeding +20 mV due to the presence of protonated amino groups on the polymer surface. Des Bouillons-Gamboa et al. [44] reported strongly positive zeta potentials ranging from +45 to +61 mV, which were considered sufficient to ensure electrostatic stabilization and prevent aggregation. Similarly, highly positive zeta potential values for TPP-crosslinked CS nanoparticles were reported in many studies [50,51,58]. The near-neutral zeta potential observed in the present study may be attributed to extensive ionic interaction between the positively charged amino groups of chitosan and the negatively charged phosphate groups of TPP, leading to partial charge neutralization at the nanoparticle surface. Although low absolute zeta potential values are generally associated with reduced electrostatic stabilization, formulation F8 maintained a very small particle size and narrow size distribution. This suggests that other stabilizing mechanisms, such as efficient crosslinking, compact nanoparticle structure, and steric or hydration-based stabilization, may have contributed to maintaining colloidal stability. In this context, CS nanoparticles may behave as lyophilic colloidal systems, in which stability is not governed solely by surface charge but also by the formation of a protective solvent layer around the particles [41]. Therefore, despite the low zeta potential values, the optimized formulation remained physically stable, indicating that electrostatic repulsion was not the only determinant of nanoparticle stability. Similar conclusions were reported by Rampino et al. [55], who emphasized that formulation composition, polymer–crosslinker interactions, and processing conditions play key roles in determining the stability of chitosan-based nanoparticles.

TEM analysis provided direct morphological confirmation of the successful formation of CIP-loaded CS nanoparticles. The particles appeared predominantly spherical, discrete, and smoothly surfaced, with the optimized formulations showing more uniform dispersion than the formulations that produced larger particles or broader size distributions. This observation supports the dynamic light scattering (DLS) findings, but it also provides complementary information because DLS reports the hydrodynamic diameter of hydrated particles in suspension, whereas TEM directly visualizes the dried particulate structure. The spherical morphology is consistent with the expected self-assembly mechanism of ionotropic gelation, in which protonated amino groups of chitosan interact electrostatically with the phosphate groups of TPP to form compact crosslinked nanostructures. Therefore, the well-defined morphology of F8 and the nanosized particles observed by TEM indicate that the optimized preparation variables were suitable for producing compact carriers capable of maintaining CIP within the CS matrix. This morphology is particularly relevant for antibacterial delivery because small, regularly shaped particles provide a high surface area and may improve the probability of contact between the drug-loaded carrier and bacterial cell envelopes.

The in vitro release findings further demonstrate the functional advantage of entrapping CIP within the CS nanoparticle matrix. The optimized formulation F8 exhibited a biphasic pattern, with an initial release during the first 12 h followed by prolonged release up to 220 h. The early phase can be explained by the release of CIP adsorbed on, or weakly associated with, the nanoparticle surface, while the later phase is likely governed by gradual diffusion of drug molecules from the ionically crosslinked CS-TPP network, together with progressive hydration and relaxation of the polymeric matrix. This interpretation was further supported by mathematical release-kinetic analysis, in which the Higuchi model provided the best fit (R^2^ = 0.979), compared with the Hixson–Crowell (R^2^ = 0.945) and zero-order (R^2^ = 0.930) models, indicating that diffusion is the principal mechanism controlling CIP release. The relatively high fit to the Hixson–Crowell model suggests that changes in particle dimensions or gradual matrix relaxation/dissolution may also contribute to the release process. Furthermore, the Korsmeyer–Peppas exponent (n = 0.334) indicates a predominantly quasi-Fickian diffusion mechanism, further supporting diffusion-controlled drug transport from the nanoparticle matrix. FSuch biphasic behavior is commonly reported for polymeric nanoparticle systems and is advantageous because it combines early drug availability with extended maintenance of antimicrobial exposure. In agreement with this interpretation, Badran et al. reported that CS modification of polymeric nanoparticles reduced the burst effect and slowed drug release by adding an additional diffusional barrier, highlighting the role of CS-containing matrices in controlled release [59]. For antibacterial therapy, this sustained release pattern is important because maintaining CIP concentrations for longer periods may support time-above-inhibitory exposure at the infection site, reduce rapid drug depletion, and contribute to the dose-sparing potential proposed for the optimized formulation.

The antibacterial results confirm that nanoparticle encapsulation translated into a meaningful pharmacodynamic benefit. CIP-loaded CS nanoparticles consistently reduced MIC and MBC values compared with free CIP across susceptible and resistant isolates. The improvement was particularly evident against MRSA-60, where MIC/MBC values decreased from 16/32 mg/L for free CIP to 4/8 mg/L for the nanoparticle formulation, and against *P. aeruginosa* PA-10 and PA-135, where both inhibitory and bactericidal values were reduced despite the resistant phenotype. This enhancement is likely multifactorial. First, the nanoscale size and spherical morphology may increase local contact between the formulation and bacterial surfaces. Second, CS can contribute intrinsic antibacterial activity through interaction with negatively charged bacterial membranes, leading to membrane disruption and leakage of intracellular components. Third, the sustained release behavior can maintain CIP availability in the bacterial microenvironment for an extended duration. Orellano et al. reported that chitosan nanoparticles showed stronger antimicrobial activity than native CS against mastitis pathogens, caused bacterial membrane damage, and inhibited biofilm formation [60]. Similarly, CS nanoparticles loaded with Ocimum basilicum essential oil damaged bacterial membranes and caused leakage of biological macromolecules while improving antibacterial and antibiofilm activity against *E. coli* and *S. aureus* [48]. The additional evidence from green-synthesized CS nanoparticles against multidrug-resistant biofilm-forming *Acinetobacter baumannii* further supports the broader role of small, positively charged or surface-active CS nanoparticles in strengthening antibacterial efficacy [52]. Although the near-neutral zeta potential recorded in the present formulations suggests that strong electrostatic stabilization was not the dominant colloidal mechanism, the combined effects of nanoscale morphology, chitosan-bacterial surface interaction, and prolonged CIP release provide a reasonable explanation for the observed 2–4-fold improvement in antibacterial activity.

The enhanced antibacterial activity of CIP-loaded CS nanoparticles is likely attributable to several complementary mechanisms. Their nanoscale dimensions and high surface area may promote closer contact and adsorption at bacterial surfaces, while the protonated amino groups of chitosan can interact with negatively charged components of the bacterial envelope, increasing membrane permeability and promoting leakage of intracellular constituents [60,61]. Such membrane perturbation and close nanoparticle–cell association may facilitate local delivery and intracellular access of the encapsulated antibiotic. Indeed, enhanced intracellular delivery and antibacterial activity of antibiotic-loaded CS nanoparticles have previously been demonstrated against intracellular bacterial pathogens [29]. In addition, encapsulation within a polymeric CS matrix may provide physical protection of the incorporated drug from premature degradation or loss before its release, which is a recognized advantage of CS-based nanoparticulate delivery systems [62]. Finally, the sustained-release behavior of the CS–TPP matrix may prolong CIP availability in the bacterial microenvironment and help maintain inhibitory drug concentrations for extended periods. Previous CIP-loaded CS nanoparticle studies have similarly demonstrated prolonged drug release together with lower MIC values than free CIP [30]. Collectively, enhanced bacterial surface interaction, membrane perturbation, improved local and potentially intracellular drug availability, protection of the encapsulated drug, and sustained antimicrobial exposure may contribute to the observed reductions in MIC and MBC. However, intracellular CIP accumulation and protection from degradation were not directly quantified in the present study and are therefore proposed as plausible contributing mechanisms based on previously reported behavior of CS-based nanoparticle systems.

Although CIP-loaded CS nanoparticles prepared by ionic gelation have been previously reported in several studies [29,63,64], the present study extends this approach through systematic optimization of the CIP–CS–TPP composition to obtain exceptionally small, highly monodisperse nanoparticles with high drug entrapment efficiency. More importantly, the optimized formulation was evaluated not only against susceptible organisms but also against clinically relevant CIP-resistant MRSA and *P. aeruginosa* isolates using both MIC and MBC endpoints. Thus, the novelty of this work lies in integrating optimized physicochemical properties, prolonged drug release, physical stability, and quantitative demonstration of enhanced inhibitory and bactericidal activity against resistant bacterial phenotypes.

Overall, the findings demonstrate that careful optimization of formulation composition and processing parameters is essential for producing stable, homogeneous, and highly efficient CIP-loaded CS nanoparticles. The superior characteristics of F8, including its nanoscale size, exceptional homogeneity, and high drug entrapment efficiency, support its selection as the lead formulation for subsequent stability, release, and antibacterial evaluations.

## 4. Materials and Methods

### 4.1. Materials

Ciprofloxacin was provided as a gift from Tabuk Pharmaceutical Company (Tabuk, Saudi Arabia). Chitosan (CS); low molecular weight (7464 g/mole), degree of deacetylation ≥ 75%, Sodium tripolyphosphate (TPP), nutrient agar pre-poured Petri dishes, Mueller–Hinton agar (cation-adjusted), were obtained from Sigma-Aldrich (St. Louis, MO, USA). All other reagents and chemicals used were of either HPLC or analytical grade.

### 4.2. Preparation of Ciprofloxacin-Loaded Chitosan Nanoparticles

CIP-loaded CS nanoparticles were prepared using the ionic gelation method with TPP as a cross-linking agent, as previously described with minor modifications [65]. Briefly, low-molecular-weight chitosan (7.464 kDa; degree of deacetylation 75%) was dispersed in distilled water and solubilized using 5 mol% acetic acid, calculated relative to the amino groups of CS. The acidic environment promotes protonation of the primary amino groups of CS, which is required for their subsequent electrostatic interaction with the negatively charged phosphate groups of TPP. CIP was dissolved separately in 2 mL of dilute hydrochloric acid and then added to the CS solution to obtain final drug concentrations ranging from 0.1 to 0.6 mg/mL, according to the formulation design shown in Table 1.

A TPP stock solution (5 mg/mL) was prepared in double-distilled water and diluted to the required working concentrations ranging from 0.1 to 1 mg/mL. Prior to nanoparticle preparation, the CS-CIP and TPP solutions were filtered through 0.45 µm and 0.22 µm membrane filters, respectively. Nanoparticles were formed by adding the TPP solution dropwise to the CS-CIP solution at room temperature under continuous magnetic stirring at 750 rpm. The interaction between the positively charged amino groups of CS and the negatively charged phosphate groups of TPP resulted in spontaneous ionic crosslinking and nanoparticle formation. Polymer-to-crosslinker ratios were adjusted between 1:1 and 2:1, depending on the formulation composition. The TPP addition procedure, mixing conditions, and temperature were maintained consistently among formulations to minimize variations in nanoparticle nucleation, crosslinking, and growth. The kinetics of ionic crosslinking were not independently quantified in the present study.

The resulting nanoparticle suspensions were centrifuged at 15,000 rpm for 30 min to collect the nanoparticle pellets. The pellets were washed twice with distilled water to remove unentrapped ciprofloxacin and excess formulation components. The purified nanoparticles were then freeze-dried for further characterization and evaluation. For one selected formulation, sonication was applied for 15 min in an ice bath at 50% amplitude to investigate the effect of sonication on nanoparticle formation and size reduction.

### 4.3. Particle Size and Zeta-Potential

Particle size (PS) and polydispersity index (PDI) of the prepared nanoparticles were determined using a ZetaPALS analyzer (Brookhaven Instruments Corporation, Holtsville, NY, USA). Prior to measurement, each sample was diluted with distilled water to achieve a nanoparticle concentration of approximately 0.1%. Analyses were conducted at a fixed scattering angle of 90°. Zeta potential measurements were carried out at 25 °C using the same instrument operated in Laser Doppler Velocimetry mode. All results were expressed as mean ± standard deviation.

### 4.4. Drug Entrapment Efficiency

To determine the amount of unentrapped CIP, an aliquot of the supernatant collected after both the first and the second centrifugation step of each CIP-loaded CS nanoparticle formulation was diluted with methanol, re-filtered, and analyzed using a previously reported spectrophotometric method [66]. The drug entrapment efficiency percentage (EE%) was then calculated using the following equation:

(1) $$EE\%=\frac{\left(W_{total\;drug}\;-\;W_{free\;drug}\right)}{W_{total\;drug}}\;\times \;100$$

### 4.5. Transmission Electron Microscopy (TEM) Imaging and Structural Analysis

The morphology and structural features of the CIP-loaded CS nanoparticles were examined using transmission electron microscopy (TEM). Briefly, an accurately weighed amount of the lyophilized nanoparticle formulation was reconstituted in distilled water to obtain a final concentration of 1 mg/L. The dispersion was then sonicated for 10 min using a bath sonicator to ensure proper redispersion of the nanoparticles and minimize aggregation. A small drop of the diluted suspension was placed onto a 400-mesh carbon-coated copper grid and allowed to air-dry at room temperature. The prepared grids were examined using a transmission electron microscope (JEM-1400, JEOL, Tokyo, Japan) operated at an accelerating voltage of 120 kV to assess nanoparticle shape, surface characteristics, and overall morphology.

### 4.6. Evaluation of Physical Stability

The physical stability of the optimized formulation (F8) was assessed after redispersion in distilled water. The nanoparticle suspension was stored under refrigerated conditions at 2–8 °C for up to 28 days. At predetermined time points, specifically after 14 and 28 days of storage, samples were withdrawn and analyzed for particle size and polydispersity index (PDI) to evaluate changes in nanoparticle size distribution and physical stability over time.

### 4.7. In Vitro Release Profile Study

The in vitro release of CIP from the prepared nanoparticles was evaluated using the dialysis bag method, as previously reported with minor modifications [67]. Briefly, accurately weighed samples from each formulation, equivalent to 1 mg of CIP, were dispersed in 1 mL of phosphate-buffered saline (PBS, pH 7.4). The dispersion was then transferred into regenerated cellulose dialysis bags with a molecular weight cut-off of 12–14 kDa.

The sealed dialysis bags were immersed in 40 mL of PBS (pH 7.4) and maintained at 37 ± 0.5 °C in a shaking water bath (SW22, Julabo, Allentown, PA, USA) operated at 80 rpm. At predetermined time intervals, 2 mL aliquots were withdrawn from the release medium and replaced immediately with an equal volume of fresh pre-warmed PBS to maintain sink conditions. The amount of CIP released into the medium was quantified using UV–visible spectrophotometry.

#### Analysis of CIP Release Kinetics

The in vitro CIP release data were analyzed by fitting the release profiles to four commonly used kinetic models: zero-order (Equation (2)), Higuchi (Equation (3)), Hixson–Crowell (Equation (4)), and Korsmeyer–Peppas (Equation (5)).

Q = k_z_ · t (2)

Q = k_h_ · t^1/2^ (3)

(100 − Q)^1/3^ = 100^1/3^ − k_hc_ · t (4)

M_t_/M^∞^ = k_p_ · t^n^ (5)

where Q represents the cumulative percentage of CIP released at time t; k_z_ is the zero-order release rate constant; k_h_ is the Higuchi diffusion release rate constant; K_hc_ is the Hixson–Crowell release rate constant. In the Korsmeyer–Peppas model, M_t_/M^∞^ represents the fraction of drug released at time t; k_p_ is the release rate constant and n is the release exponent used to characterize the drug-release mechanism. The value of n was determined from the slope of the linear plot of log (M_t_/M^∞^) versus log t, using release data corresponding to M_t_/M^∞^ ≤ 0.6.

### 4.8. Evaluation of Antibacterial Activity

#### 4.8.1. Bacterial Strains and Culture Conditions

The antibacterial activity of the CIP-loaded CS nanoparticles was evaluated against a panel of bacterial strains, including ATCC reference strains and clinical isolates representing both Gram-positive and Gram-negative bacteria. Bacterial stocks stored in glycerol at −40 °C were revived by streaking onto Luria–Bertani (LB) agar plates, followed by incubation at 37 °C for 18–20 h to obtain fresh, isolated colonies. For susceptibility testing, bacterial cultures were prepared in Mueller–Hinton Broth (MHB), while Mueller–Hinton Agar (MHA) plates were used for bactericidal evaluation. All experiments were performed under standardized culture and incubation conditions.

#### 4.8.2. Antibacterial Testing

- Minimum Inhibitory Concentration (MIC)

The MIC values were determined using the broth microdilution method in sterile 96-well plates, following EUCAST/ESCMID (2003) guidelines [68] with minor modifications. Briefly, 100 µL of MHB was added to each well, and two-fold serial dilutions of the CIP-loaded nanoparticle formulations were prepared across the plate. The concentration range tested for the CIP-loaded formulation was 12.5–0.39 µg/mL, whereas free CIP was evaluated over a concentration range of 32–1 µg/mL. Bacterial suspensions were adjusted to a 0.5 McFarland standard using 0.9% saline and subsequently diluted in MHB to achieve a final inoculum of approximately 5 × 10^5^ CFU/mL in each well. Sterility controls containing MHB only and growth controls containing MHB with bacterial inoculum were included in each assay. The plates were incubated at 37 °C with shaking at 90 rpm for 18–20 h. The MIC was defined as the lowest concentration that completely inhibited visible bacterial growth.

- Minimum Bactericidal Concentration (MBC)

Following MIC determination, 1 µL aliquots from wells showing no visible bacterial growth were streaked onto MHA plates and incubated at 37 °C for an additional 18–20 h. The MBC was defined as the lowest concentration of the CIP formulation that resulted in at least 99% bacterial killing compared with the initial inoculum.

## 5. Conclusions

This study successfully developed and optimized CIP-loaded CS nanoparticle using a simple ionic gelation approach. The main achievement of this work was the identification of an optimized formulation capable of producing highly uniform nanosized particles with excellent drug entrapment, favorable morphology, acceptable physical stability, and sustained CIP release. Among the investigated formulations, F8 demonstrated the most promising physicochemical profile, with a remarkably small particle size, narrow size distribution, and high encapsulation efficiency, confirming the critical role of formulation composition and polymer-to-crosslinker balance in controlling nanoparticle performance.

The optimized nanoparticles exhibited a biphasic drug release pattern, combining an initial release phase that may support rapid antibacterial action with a prolonged release phase that can maintain CIP availability over an extended period. This controlled-release behavior represents an important advantage over free CIP, as it may reduce rapid drug depletion and support sustained antimicrobial exposure at the infection site.

Importantly, CIP encapsulation within CS nanoparticles translated into improved antibacterial activity. The optimized formulation reduced both MIC and MBC values compared with free CIP, particularly against CIP-resistant methicillin-resistant *Staphylococcus aureus* and *Pseudomonas aeruginosa* clinical isolates. This enhancement is likely attributed to the combined effects of nanoscale particle size, improved interaction with bacterial surfaces, intrinsic CS-related antibacterial properties, and sustained CIP release.

Overall, the findings demonstrate that optimized CIP-loaded CS nanoparticles represent a promising nanocarrier platform for improving CIP delivery and strengthening its activity against resistant bacterial strains. This formulation provides a potential dose-sparing strategy and a valuable approach for repurposing conventional antibiotics to address antimicrobial resistance. Further in vivo, pharmacokinetic, toxicity, and infection-model studies are warranted to confirm the translational potential of this optimized nanoparticle system.

Although antibiofilm, bacterial adhesion, mature-biofilm eradication, and intracellular antibacterial activities were not directly evaluated, these aspects warrant further investigation. Future studies may also explore multifunctional and ligand-targeted CS nanoparticles, biofilm-responsive systems, AI-assisted formulation optimization, and combination approaches with antimicrobial peptides or bacteriophages to further enhance efficacy against persistent and multidrug-resistant infections.

## Figures and Tables

**Table 1:** The composition of the CIP-Loaded CS nanoparticles formulations.

Formula	CIP Conc. (mg/mL)	CS Conc. (mg/mL)	TPP Conc. (mg/mL)	Ratio CS:TPP	Sonication
F1	-	0.3 mg/mL	0.1 mg/mL	1:1	No
F2	-	0.5 mg/mL	0.5 mg/mL	1:1	Yes
F3	-	0.5 mg/mL	0.5 mg/mL	2:1	No
F4	-	0.5 mg/mL	1 mg/mL	1:1	No
F5	-	1 mg/mL	0.5 mg/mL	1:1	No
F6	0.3 mg/mL	0.3 mg/mL	0.5 mg/mL	1:1	No
F7	0.6 mg/mL	0.3 mg/mL	0.5 mg/mL	1:1	No
F8	0.15 mg/mL	0.3 mg/mL	0.5 mg/mL	1:1	No
F9	0.1 mg/mL	0.3 mg/mL	0.5 mg/mL	1:1	No

**Table 2:** The mean ± standard deviation for formulations F6–F9 particle size, polydispersity, zeta potential, and EE%.

Formula	Particle Size ± SD	Polydispersity Index ± SD	Zeta Potential ± SD	EE (%)
F1	395.3 ± 6.09	0.317 ± 0.004	−1.68 ± 1.13	-
F2	628.5 ± 3.79	0.332 ± 0.002	3.6 ± 3.33	-
F3	479.0 ± 7.05	0.245 ± 0.013	0.36 ± 0.49	-
F4	3907.4 ± 315.35	0.33 ± 0.011	0.04 ± 0.55	-
F5	1431.8 ± 109.65	0.482 ± 0.017	0.62 ± 0.47	-
F6	58.09 ± 0.30	0.298 ± 0.001	−0.5 ± 0.75	59.1%
F7	115.07 ± 0.51	0.115 ± 0.037	0.92 ± 0.94	48.3%
F8	38.39 ± 0.63	0.011 ± 0.001	−0.35 ± 0.65	84.1%
F9	63.03 ± 23.41	1.37 ± 0.23	0.41 ± 0.32	88%

**Table 3:** Kinetic Parameters of CIP Release Fitted to Different Mathematical Models.

Formulation Code	Zero-Order	Higuchi Diffusion	Hixson–Crowell	Peppas–Korsmeyer Exponent (n)
F8	0.930	0.979	0.945	0.334

**Table 4:** Minimum inhibitory concentration (MIC) and minimum bactericidal concentration (MBC) of free ciprofloxacin (CIP) and ciprofloxacin nanoparticles (CIP-NPs).

Bacteria	AST	Free CIP (mg/L)		CIP NPs (mg/L)	
		MIC	MBC	MIC	MBC
*Escherichia coli* ATCC 25922	Susceptible	0.06	0.125	<0.06	≤0.06
Methicillin-resistant *S. aureus* MRSA-60 *	Resistant	16	32	4	8
MRSA-145 *	Resistant	8	16	4	8
*Pseudomonas aeruginosa* PA-10 *	Resistant	32	64	16	16
*P. aeruginosa* PA-135 *	Resistant	64	128	32	32
*P. aeruginosa* PA-598 *	Susceptible	0.25	0.5	0.125	0.25

* Clinical isolates. Abbreviations: AST, antibacterial susceptibility testing. CIP, ciprofloxacin. NPs, Nanoparticles. MIC, minimum inhibitory concentration. MBC, minimum bactericidal concentration. AST profile was determined using the European Committee on Antimicrobial Susceptibility Testing. Delafloxacin Rationale Document, version 2.0, 2021. http://www.eucast.org/rd.

## Data Availability

The original contributions presented in this study are included in the article. Further inquiries can be directed to the corresponding author.
